# Fatty Waste from Industrial Vegetable Oil Esterification as an Alternative Substrate for Rhamnolipid Production

**DOI:** 10.3390/microorganisms14102311

**Published:** 2026-10-10

**Authors:** João Paulo Chiconelo Sutilo de Arruda, Marcia Nitschke

**Affiliations:** São Carlos Institute of Chemistry (IQSC), University of São Paulo, Trabalhador São-Carlense Av., 400, São Carlos 13566-590, SP, Brazil; jpaulocsa@gmail.com

**Keywords:** biosurfactant, esterification fatty waste, rhamnolipid, *P. aeruginosa*

## Abstract

Biosurfactants are amphiphilic molecules produced by microorganisms that have attracted increasing interest as alternatives to conventional surfactants. However, the use of low-cost substrates is essential for improving the economic and environmental feasibility of their production. This study evaluated fatty waste from industrial vegetable oil esterification reactions as an alternative substrate for rhamnolipid (RL) production by *Pseudomonas aeruginosa* LBI. The residue, consisting of a mixture of carboxylic acids and esters, was used as a carbon source in the culture medium. After 144 h of cultivation, 97% of the residue was consumed, resulting in 9.32 g/L of RL. Analysis of the residual substrate indicated preferential utilization of free fatty acids followed by ester compounds. The RL product exhibited a critical micelle concentration (CMC) of 44.3 mg/L, while chemical characterization confirmed the presence of RhaC_10_C_10_ and Rha_2_C_10_C_10_ congeners. In detergency assays, the RL preparation promoted the removal of grease and tomato sauce soils from stainless steel and glass surfaces, providing preliminary evidence of the detergency potential of RL produced from the fatty residue. These findings demonstrate the potential of using the fatty residue from the chemical esterification process as a feedstock for RL production and highlight its value for waste valorization and the development of sustainable cleaning formulations.

## 1. Introduction

Growing regulatory and consumer demand for sustainable practices are driving industrial sectors toward alternatives to synthetic compounds. In this context, microbial surfactants, or biosurfactants (BS), have attracted increasing attention because of their biological origin, biodegradability, low toxicity, and production from renewable resources [1,2]. In addition to reducing surface and interfacial tension and stabilizing emulsions, BS can exhibit antimicrobial, antibiofilm, antioxidant, anticancer, and immunomodulatory activities [3], supporting their application in agriculture, environmental remediation, pharmaceuticals, food, cosmetics, and household products. This increasing scientific and industrial interest is reflected in the continued expansion of the global BS market, highlighting their potential as sustainable alternatives to conventional surfactants [4].

Among microbial BS, rhamnolipids (RLs) produced by *Pseudomonas* species, particularly *Pseudomonas aeruginosa*, have been extensively investigated because of their high surface and interfacial activity, as well as their techno-economic viability [5]. Structurally, RLs are glycolipids consisting of a polar moiety containing one or two rhamnose units, corresponding to mono- and di-rhamnolipids, respectively, linked to saturated or unsaturated β-hydroxy fatty acid chains of variable length [6]. RLs are produced as mixtures of structural congeners, whose composition can influence their physicochemical and biological properties [7]. Their low critical micelle concentrations and ability to effectively reduce surface and interfacial tension have contributed to growing interest in their application across several industrial fields [6].

Despite these favorable properties, the large-scale production of RLs remains constrained by their relatively high production costs. Raw materials, particularly the carbon source, represent an important contributor to overall production costs, making the use of inexpensive and renewable feedstocks, including industrial by-products and agro-industrial residues, an important strategy for improving the economic feasibility of RL production [8,9]. Adding value to waste streams can additionally improve the environmental and economic sustainability of BS production processes. Consequently, considerable research has focused on replacing refined substrates with alternative low-cost feedstocks. Such strategies can contribute to the circular bioeconomy by converting low-value residues into value-added biobased products and reducing the environmental burden associated with waste disposal [10].

Lipid-rich residues are particularly attractive substrates for RL production because fatty acids and related compounds can serve as carbon sources for *Pseudomonas* growth and biosurfactant synthesis [11]. Several lipid-containing residues, including used cooking oils, soapstocks, fatty acid distillate, and fatty acid methyl esters, have been investigated for RL production [12,13,14], demonstrating the feasibility of converting low-value lipid streams into biosurfactants. However, the suitability of lipid-rich substrates depends strongly on their chemical composition, degree of hydrolysis and esterification, fatty acid profile, and industrial origin, which can affect substrate assimilation and RL productivity [12,15].

Esterification is widely employed in the oleochemical and biofuel industries to convert fatty acids and alcohols into esters with commercial applications. Depending on the raw materials and processing conditions, industrial ester production can generate effluents containing residual lipid-derived compounds, including unreacted fatty acids and esters. Subsequent neutralization and acidification of these effluents can promote the separation of lipid-rich fractions containing long-chain carboxylic acids and esterified compounds [16]. Although these fractions are generally regarded as low-value industrial residues, their lipid composition indicates that they could serve as alternative carbon sources for microbial processes, including RL production.

In this context, lipid-rich waste recovered from the treatment of effluents generated during industrial ester production represents a potentially valuable and relatively unexplored feedstock for RL production. Unlike conventional vegetable oils, these residues may contain complex mixtures of free long-chain fatty acids and esters, with their composition depending on the raw materials and processing conditions employed during ester production. Such compositional differences may affect substrate utilization, microbial growth, RL production, and the composition of the resulting biosurfactant [12,17]. Thus, evaluating these residues as individual feedstocks is important for determining their suitability for RL production and for understanding how their chemical composition influences the bioprocess. At the same time, their use as fermentation substrates could contribute to the valorization of an industrial waste stream within a circular production strategy.

Detergency is of particularly interest because the surface-active properties of BS make them candidates for cleaning applications, and previous studies have demonstrated their ability to facilitate the removal of hydrophobic compounds [18,19]. Evaluating the detergency of RLs produced from industrial residues therefore provides an additional level of validation, helping to determine whether substrate valorization can generate BS with practical functional value.

Based on these considerations, the present study investigated the feasibility of using a lipid-rich residue recovered from a neutralization effluent generated by an industrial ester-production process as an alternative carbon source for RL production by *Pseudomonas aeruginosa*. The resulting RLs were characterized and their detergency performance was evaluated using a model fat-based cleaning assay.

## 2. Materials and Methods

### 2.1. Microorganism

The rhamnolipid-producing strain *Pseudomonas aeruginosa* LBI [20] was maintained at −20 °C and activated on nutrient agar prior to the experiments.

### 2.2. Culture Media

For strain maintenance, nutrient agar plates were prepared using nutrient broth (8 g/L; Neogen Culture Media, Lansing, MI, USA) supplemented with agar (20 g/L; Kasvi, Curitiba, Brazil) with the pH adjusted to 6.8.

Rhamnolipid production and inoculum preparation was carried out using a mineral medium adapted from Robert et al. [21] containing (g/L) NaNO_3_ (4.0), KCl (0.1), KH_2_PO_4_ (0.5), K_2_HPO_4_ (1.0), CaCl_2_ (0.01), MgSO_4_·7H_2_O (0.5), FeSO_4_·7H_2_O (0.01), yeast extract (0.1) and 0.05 mL/L of trace element solution. The trace element solution contained (g/L) H_3_BO_3_ (1.5), CuSO_4_·5H_2_O (2.0), MnSO_4_·H_2_O (1.5), (NH_4_)_6_Mo_7_O_24_·4H_2_O (0.1), and ZnSO_4_·H_2_O (1.9). The final pH was adjusted to 6.8. The medium was supplemented with 2% (*w*/*v*) esterification-derived fatty residue as the carbon source, as previously described [20], and sterilized by autoclaving at 121 °C for 15 min.

### 2.3. Esterification-Derived Fatty Residue

The alternative carbon source was obtained from a fatty residue generated during the industrial esterification process at Capuani do Brasil S/A (São Paulo, Brazil). This industrial process produces a neutralization effluent containing water, inorganic salts, fatty acids, and esters. To recover the lipid fraction, a mixture of effluents from different industrial batch processes was acidified with concentrated hydrochloric acid until pH 3.0. After phase separation by decantation, the upper lipid fraction was collected and used as an alternative carbon source for RL production. The resulting residue was analyzed by gas-chromatography, and its composition is shown in Table 1.

### 2.4. Inoculum Preparation

The *P. aeruginosa* LBI strain was transferred from frozen stocks to nutrient agar plates and incubated at 30 °C for 24 h. Subsequently, two loops of bacterial biomass were suspended in 8 mL of sterile 0.85% (*w*/*v*) NaCl solution and homogenized using a vortex mixer. The cell suspension was adjusted to an optical density of 0.65 at 610 nm, corresponding to approximately 1 × 10^8^ CFU/mL, using a UV–Vis spectrophotometer (Genesys 10UV Scanning, Thermo Scientific, Waltham, MA, USA). An aliquot of 1 mL was then inoculated into 125 mL Erlenmeyer flasks containing 25 mL of mineral medium supplemented with fatty residue. The inoculum cultures were incubated for 24 h at 30 °C and 180 rpm in a rotary shaker (TE-420, Tecnal, Piracicaba, Brazil).

### 2.5. Rhamnolipid Production

RL production experiments were performed in 125 mL Erlenmeyer flasks containing 25 mL of mineral medium (2% fatty residue as carbon source) and 1 mL of the bacterial inoculum prepared as described above. The cultures were incubated for 144 h at 30 °C and 200 rpm in a shaker incubator (Innova 43, New Brunswick, Enfield, CT, USA). All experiments were performed in triplicate. Samples were periodically collected throughout the cultivation period for evaluation of cell growth and surfactant production parameters.

### 2.6. Analytical Methods

#### 2.6.1. Sample Processing

Culture samples were transferred to centrifuge tubes and extracted with 20 mL of hexane. The samples were vortexed and centrifuged at 10,000 rpm for 15 min at 4 °C (Legend RT+, Thermo Scientific, Waltham, MA, USA) to separate the organic and aqueous phases. The organic phase was used for quantification of residual carbon source, whereas the aqueous phase was analyzed for pH, surface tension, critical micelle dilution (CMD), and rhamnolipid content, as described below.

#### 2.6.2. Microbial Growth

An aliquot of 1 mL of the whole culture medium (before centrifugation) was submitted to serial dilution and viable cell counts determined by the drop plate method [22].

#### 2.6.3. pH Measurement

pH of the aqueous phase obtained after sample processing was measured using a digital pH meter (W38 pHmeter, BEL, São Paulo, Brazil).

#### 2.6.4. Rhamnolipid Quantification

RL present in culture medium and the extracted rhamnolipids were analyzed by high-performance liquid chromatography coupled with evaporative light scattering detection (HPLC–ELSD) (Thermo Scientific, Waltham, MA, USA). Analyses were performed using an Agilent 1260 Infinity HPLC system equipped with an ELSD detector (Agilent, Santa Clara, CA, USA) set at 60 °C, a nitrogen pressure of 3.5 bar, and gain 1. Separation was achieved using a reverse-phase Zorbax Eclipse Plus RR C18 column (4.6 × 100 mm, 3 µm) maintained at 30 °C. A linear gradient elution was applied using 0.1% (*w*/*v*) formic acid in ultrapure water and acetonitrile as mobile phases. The acetonitrile concentration was increased from 50 to 80% over 15 min at a flow rate of 1 mL/min [23]. RL concentration was calculated from external calibration using a commercial standard (Rhamnolipids R90, Sigma-Aldrich, St. Louis, MO, USA); therefore, the reported values should be interpreted as R90-equivalent concentrations.

#### 2.6.5. Quantification of Fatty Residue

Fatty compounds present in medium were extracted with hexane and analyzed by gas chromatography coupled with flame ionization detection (GC-FID). Analyses were performed using a GC-HP 6850 system (Agilent, Santa Clara, CA, USA) equipped with an INNOWAX capillary column (30 m × 0.25 mm × 0.25 µm). Helium was used as carrier gas at a flow rate of 1.5 mL min^−1^ (37 cm/s). The injector and detector temperatures were set at 250 and 280 °C, respectively, with a split ratio of 40:1. The oven temperature program started at 120 °C and was maintained for 1 min, followed by heating at 10 °C/min to 200 °C (maintained for 3 min) and subsequently to 250 °C (maintained for 5 min), according to Guan et al. [24]. The consumption of fatty compounds was determined based on peak areas and quantified using external calibration curves prepared from isolated fatty acid and ester standards.

### 2.7. Recovery of Rhamnolipids

RL was extracted using an aqueous two-phase system composed of ammonium sulfate and 2-propanol, according to Zhang et al. [25]. Briefly, 8 mL of cell-free culture broth was transferred to a 15 mL Falcon tube, followed by the addition of 20% (*w*/*v*) ammonium sulfate and 50% (*v*/*v*) 2-propanol, and the pH of the mixture was adjusted to 5.0 using sulfuric acid (5 mol/L). The tube was vortexed for 30 s and maintained at room temperature for 60 min to allow phase separation. The alcohol phase was separated, supplemented with 8 mL of 2-propanol and concentrated by rotary evaporation (Rotavapor R-100, Büchi, Flawil, Switzerland) to obtain the RL product.

### 2.8. Rhamnolipid Characterization

#### 2.8.1. Fourier Transform Infrared Spectroscopy (FTIR) Analysis

The obtained RL were characterized by attenuated total reflectance Fourier transform infrared spectroscopy (ATR-FTIR) using a Cary 630 spectrometer (ATR diamond single-bounce, Agilent, Santa Clara, CA, USA). A small amount of dried rhamnolipid extract was directly placed onto the ATR crystal, and spectra were recorded. The obtained profiles were compared with those of a commercial rhamnolipid standard (Rhamnolipids R90, Sigma-Aldrich, St. Louis, MO, USA).

#### 2.8.2. Mass Spectrometry

The molecular composition of the extracted rhamnolipids was analyzed by electrospray ionization time-of-flight mass spectrometry (ESI-TOF-MS). Rhamnolipid extracts were dissolved in methanol at 1 mg/mL and directly introduced into a micrOTOF mass spectrometer (Bruker, Ettlingen, Germany). Analyses were performed using a capillary voltage of 4 kV, nitrogen gas temperature of 180 °C, pressure of 2.0 bar, and flow rate of 8.0 L/min. Spectra were acquired in negative ion mode over an *m*/*z* range of 50–1500 [26].

#### 2.8.3. Surface and Interfacial Tension Measurements

Surface tension, critical micelle dilution (CMD), and critical micelle concentration (CMC) analyses were performed using a Sigma 700 tensiometer (Attension, Espoo, Finland) based on the Du Noüy ring method. For CMD determination, aqueous culture extracts were diluted 10- and 100-fold (CMD^−1^ and CMD^−2^, respectively), and surface tension measurements were performed at room temperature. For CMC determination, extracted RL solutions (0.5 g/L) were progressively diluted in distilled water using an automatic titration system and CMC further calculated using the Attension Sigma software (OneAttension v.2.8). Interfacial tension measurement between rhamnolipid solution (0.1% *w*/*v*) and hexadecane was also performed using the Du Noüy ring method.

### 2.9. Detergency Performance Evaluation

The detergency performance of the RL obtained from the esterification-derived fatty residue was evaluated in comparison with tap water, a commercial dishwashing detergent (CDD), sodium lauryl ether sulfate (SLES), and sodium dodecyl benzene sulfonate (SDBS). Cleaning solutions were prepared at a final surfactant concentration of 1% (*w*/*v*). The detergency assays were performed using two types of models: a synthetic grease mixture and tomato sauce. The synthetic grease mixture was prepared using butter, lard, and sunflower oil (1:1:1, *w*/*w*), supplemented with 0.1% (*w*/*w*) activated charcoal [27]. The grease mixture was applied at 50 °C onto stainless steel spatulas and glass slides samples and allowed to solidify at room temperature. Tomato sauce obtained from a local market was applied directly onto the sample surfaces and dried at 60 °C for 30 min. The model soils were applied to approximately one-third of the surface area of each test coupon. The average mass applied was 81.6 mg for the grease mixture and 21.3 mg for tomato sauce, with the soils distributed over the designated test area of the stainless-steel and glass surfaces. The contaminated surfaces were individually immersed in 40 mL of each cleaning solution for 10 min without agitation at room temperature, followed by rinsing in 40 mL of distilled water. Samples were dried at room temperature, and cleaning efficiency was determined by measuring the difference in mass before and after cleaning, using the following equation:

% CE = (W_c_ − W_w_)/(W_c_ − W_i_) × 100

where W_c_ is the weight of the contaminated test sample, W_w_ is weight of the test sample after washing and W_i_ is the initial weight of the test sample [28].

### 2.10. Statistical Analysis

Experiments were performed using three independent replicates, and results were expressed as mean values ± standard deviation. Analysis of variance (ANOVA), followed by the Tukey test, was performed with OriginPro software, version 9.0 (OriginLab Corporation, Northampton, MA, USA).

## 3. Results and Discussion

### 3.1. Production of RL Using Fatty Residue as a Carbon Source

**Figure 1:**
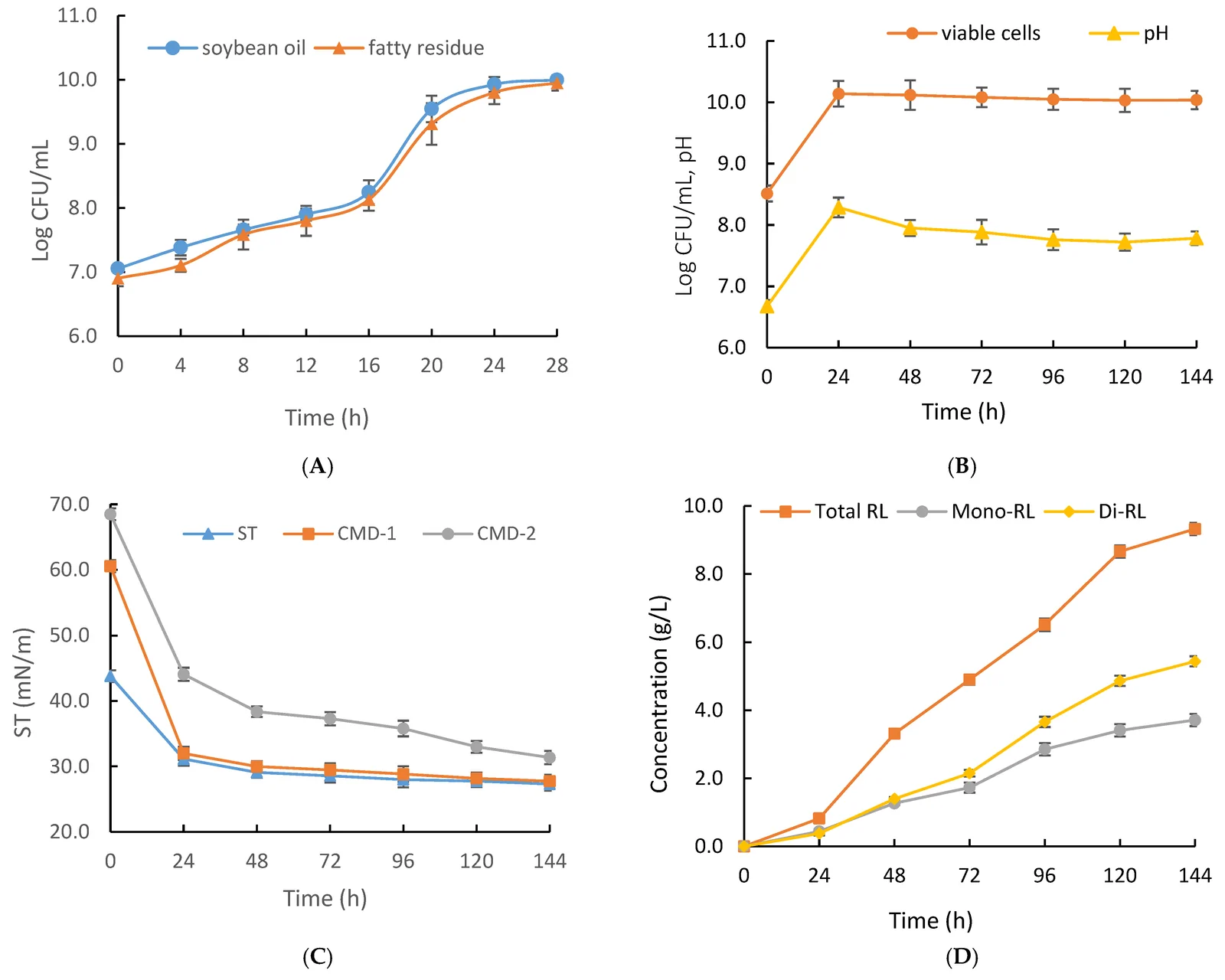
Time kinetics of RL production by *P. aeruginosa* LBI using fatty residue: (**A**) inoculum growth in fatty residue and soybean oil; (**B**) cell growth in fatty residue; (**C**) surface tension; (**D**) RL quantification as R-90 equivalent.

Analysis of the fatty residue recovered from the neutralization effluent revealed the presence of three distinct groups of compounds (Table 1): fatty acids (29.0%), methyl esters (46.8%), and other esters derived from alcohols containing more than three carbon atoms (24.2%). The suitability of this residue as an alternative carbon source for bacterial growth and RL production was then evaluated. Bacterial inoculum was prepared in mineral medium supplemented with the fatty residue, while a parallel medium, containing the same concentration of soybean oil as the carbon source [29], served as a reference for comparing growth performance. As shown in Figure 1A, *P. aeruginosa* LBI exhibited similar growth performance on both carbon sources, indicating that the strain is able to effectively grow using the residue under the tested conditions. Thus, in subsequent experiments, the inoculum was prepared in fatty residue medium cultivated for 24 h.

Further studies were conducted to evaluate the kinetic profile of rhamnolipid production in the proposed fatty residue medium. Cultivation using the alternative carbon source showed that the cell population increased during the first 24 h and remained constant until the end of the 144 h period (Figure 1B), a profile similar to that observed in previous study using sunflower oil refining waste for cultivation of *P. aeruginosa* LBI [30]. The slight increase in pH, followed by stabilization around 8.0, is consistent with a previous study reporting that growth and RL production by *P. aeruginosa* using kitchen waste oil as the sole carbon source were favored under weakly alkaline conditions [31].

The surface tension and critical micelle dilution parameters (CMD^−1^, CMD^−2^) of the culture medium decreased during the first 24 h of cultivation, reaching values of 31.1 mN/m for surface tension, 32.0 mN/m for CMD^−1^, and 44.1 mN/m for CMD^−2^ (Figure 1C). This decreasing profile persisted until the end of the process, reaching final values of 27.3 mN/m (ST), 27.8 mN/m (CMD^−1^), and 31.3 mN/m (CMD^−2^), indicating a continuous increase in biosurfactant concentration, particularly evident at the 10× and 100× dilutions of the medium.

The rhamnolipid content in the medium was quantified by HPLC-ELSD analysis. RL concentration (R90 equivalent) increased steadily throughout the process, reaching 9.32 g/L at the end of the 144 h incubation period (Figure 1D). When soybean oil was utilized as carbon source under the same conditions, the kinetics profile was similar, reaching a final RL concentration of 8.13 g/L. These RL levels are consistent with values reported for cultivation of *P. aeruginosa* LBI on various oil refinery wastes, including cottonseed (10.5 g/L), babassu (8.6 g/L), corn (13.5 g/L), palm (8.6 g/L), and soybean (11.7 g/L) residues [29]. By contrast, lower RL titers have been reported for other *P. aeruginosa* strains grown on waste-oil-based substrates: 1.1 g/L from *P. aeruginosa* M4 using waste cooking oil [32], 6.3 g/L using a waste cooking oil/glycerol combination [33], and 3.5 g/L from *P. aeruginosa* PAO1 using distilled palm fatty acids and their methyl esters [12].

Quantification of the main congeners revealed distinct production kinetics between mono- and di-rhamnolipid. At 24 h, mono-RL levels (0.442 g/L) exceeded di-RL (0.381 g/L); by 48 h, this pattern reversed, and di-RL remained the dominant congener throughout cultivation (Figure 1D). Final concentrations reached 3.71 g/L mono-RL and 5.61 g/L di-RL, corresponding to a mono/di ratio of 0.66. This shift resembles the pattern reported for *Pseudomonas aeruginosa* UFPEDA 614 grown on glycerol, where mono-RL predominated during the first 48 h before di-RL became dominant, reaching 75% of total RL after 96 h [34]. Such conversion reflects the biosynthetic pathway of RL congeners as di-RL synthesis depends on mono-RL availability, since the glycosyltransferase RhlC uses mono-RL as substrate to add a second rhamnose moiety [35]. Di-RL predominance has been widely reported in RL mixtures, with mono/di ratios of 25.5/74.5 for *P. aeruginosa* on sunflower oil [36] and 11.4/88.5 for *P. aeruginosa* KT1115 on rapeseed oil [26]. However, the synthesis of mono-RL and di-RL by *P. aeruginosa* PAL 05 using different carbon sources revealed that mono-RL predominated with hydrophobic carbon sources (myristic acid and turnip oil), while di-RL prevailed when using hydrophilic sources such as glycerol or glucose [37], as also observed in previous studies using *P. aeruginosa* LBI [29]. These findings suggest that the proportion of mono and di-RL congeners is influenced by several factors including bacterial strain, culture conditions, and type of carbon source.

**Figure 2:**
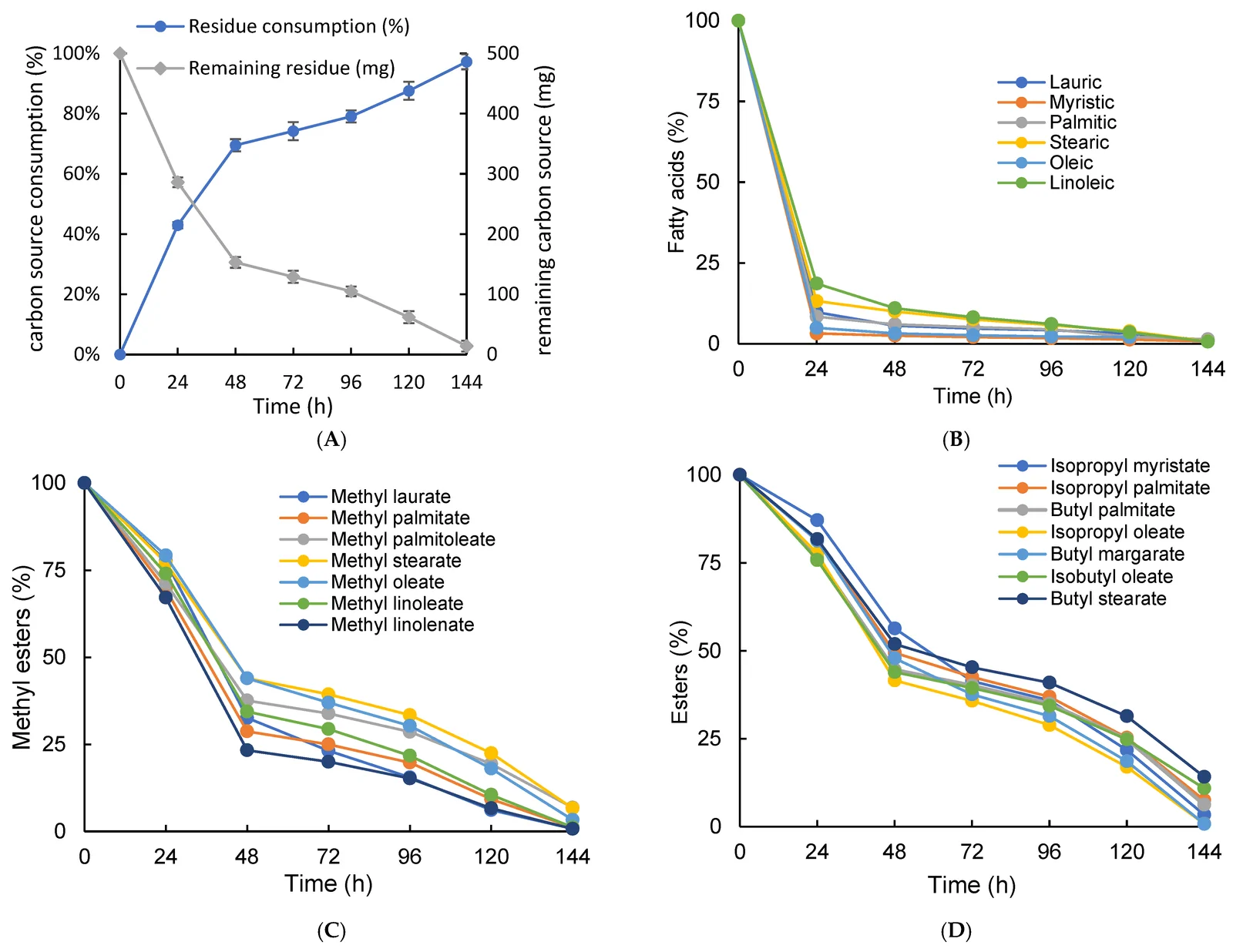
Consumption of fatty residue components during RL production by *P. aeruginosa* LBI: (**A**) total fatty residue; (**B**) fatty acids; (**C**) methyl esters; (**D**) other esters.

The carbon source consumption profile during RL production from fatty residue is shown in Figure 2. Each flask received 500 mg of the carbon source, and the highest average consumption (43%) occurred within the first 24 h. By the end of the incubation period, 97% of the fatty residue was consumed, leaving a residual mass of 14 mg (Figure 2A). Considering these results, estimated RL yield was 0.48 g RL/g substrate consumed with volumetric productivity of 0.0647 g/L/h. To evaluate whether the bacteria could utilize the individual components present in the fatty residue, consumption of each compound was analyzed throughout cultivation. Fatty acids were rapidly consumed within the first 24 h, decreasing to 5–19% relative to their initial concentrations (Figure 2B). Myristic and oleic acids showed the highest consumption rates, while stearic and linoleic acids showed the lowest. Consumption of the remaining fatty acids continued over the course of the process, and by the end of incubation, concentrations had dropped below 1%.

The fatty acid methyl esters (Figure 2C), in turn, showed 21–33% consumption in the first 24 h, following the increasing order C18:1 < C12, C18 < C18:2 < C16:1 < C16 < C18:3. Over the next 24 h, this group of compounds reached its highest consumption rate, with values between 52 and 77% and an increasing order of C18:1 < C18 < C16:1 < C18:2 < C12 < C16 < C18:3, ultimately reaching 93–99% consumption by the end of the 144 h cultivation period.

The esters derived from alcohols with three or more carbons (Figure 2D) showed consumption profiles similar to those of the methyl esters, but with lower consumption rates in the first 24 h, ranging between 13 and 24%. Over the subsequent 24 h, consumption increased to values between 44 and 56%. These esters were further consumed throughout cultivation, albeit at a lower rate, reaching 86–99% consumption by the end of the 144 h period.

Comparison of cell growth (Figure 1B) with the corresponding fatty acid consumption profile (Figure 2B) suggests that these substrates are efficiently utilized by bacterial cells, as indicated by their rapid depletion from the culture medium. In *P. aeruginosa*, free fatty acids can be transported across the outer membrane through the FadL protein channel, which facilitates their passage through the polar lipopolysaccharide layer [38]. By contrast, the utilization of ester substrates requires an additional hydrolysis step to release fatty acids that can subsequently be incorporated into cellular metabolism. Lipases are generally characterized by their ability to hydrolyze long-chain acylglycerols, whereas esterases preferentially hydrolyze esters containing shorter-chain fatty acids [39]. *P. aeruginosa* produces several lipolytic enzymes capable of hydrolyzing ester substrates. For example, Lip8 from *P. aeruginosa* LST-03 exhibited hydrolytic activity toward fatty acid methyl esters (FAMEs), particularly those containing short-chain fatty acids [40].

Another interesting observation is that ester consumption became more pronounced after 48 h, coinciding with the shift in RL congener profile. This temporal association raises the possibility that the presence of esterified substrates may contribute to the increased production of di-RL during the later stages of cultivation. Di-RL has a higher hydrophilic–lipophilic balance (HLB) than mono-RL [7], which may enhance interactions with ester-containing substrates and facilitate their accessibility to lipolytic enzymes in aqueous medium. Thus, the shift toward di-RL predominance may favor ester hydrolysis and fatty acid release for subsequent cellular uptake; however, this hypothesis remains speculative and requires further experimental validation.

The free fatty acids present in the fatty residue were consumed more rapidly by *P. aeruginosa* LBI than the methyl esters, followed by the other ester compounds. This pattern differs from that reported by Radzuan et al. [41], who found that FAMEs were completely consumed in a shorter time than distilled palm fatty acids (PFADs) when these substrates were evaluated separately for RL production by *P. aeruginosa* PAO1. The authors attributed this difference to the solid state of PFADs at the incubation temperature, whereas the corresponding methyl esters remained liquid, facilitating their assimilation by the bacterium [41]. In the present study, however, the fatty acids were part of a complex, ester-rich residue, and their coexistence with the ester fraction may have enhanced their dispersion and availability in the culture medium, potentially contributing to their more rapid consumption. This interpretation should nevertheless be considered tentative, as the individual effects of substrate phase behavior, solubility, and interactions among the different residue components were not independently evaluated.

A techno-economic assessment of di-rhamnolipid production by *Pseudomonas putida* KT2440 using glucose, glycerol, soybean oil, and stearic acid as feedstocks was reported. Stearic acid was identified as the most promising substrate, providing the highest di-rhamnolipid yield, lowest specific energy consumption, highest economic performance, and lowest environmental impact [15]. Nevertheless, the authors also highlighted the availability of vegetable oils, particularly soybean oil, as an important consideration in feedstock selection. The extensive consumption of the fatty residue observed in the present study, encompassing its major components, free fatty acids and esterified compounds, indicates that *P. aeruginosa* LBI can utilize this complex substrate to support bacterial growth and RL synthesis, highlighting its potential as an alternative carbon source for RL production. However, future studies are needed to evaluate substrate conversion efficiency and product recovery to further substantiate its suitability as a feedstock for RL production.

### 3.2. Structural and Physicochemical Characterization of BS

**Figure 3:**
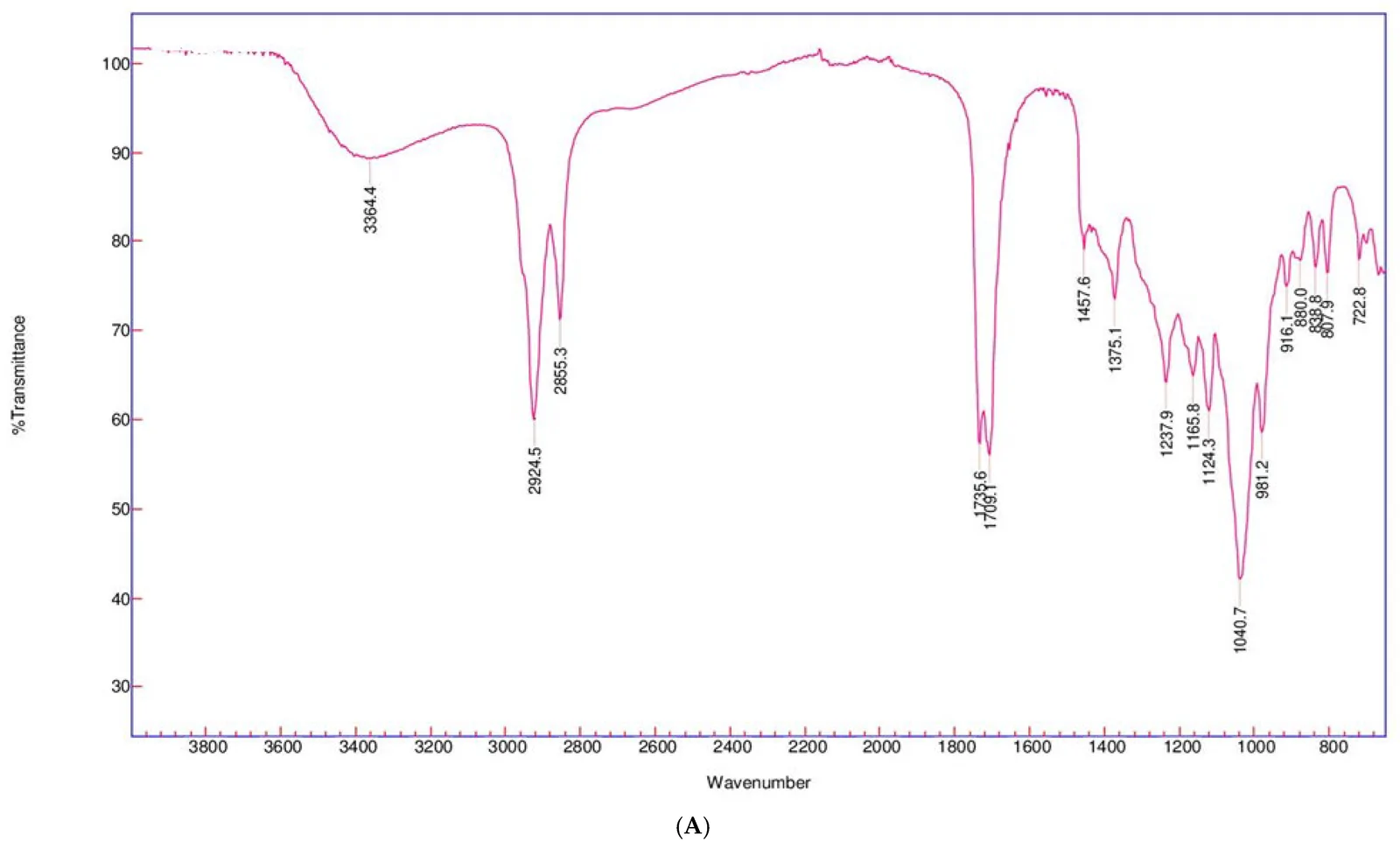

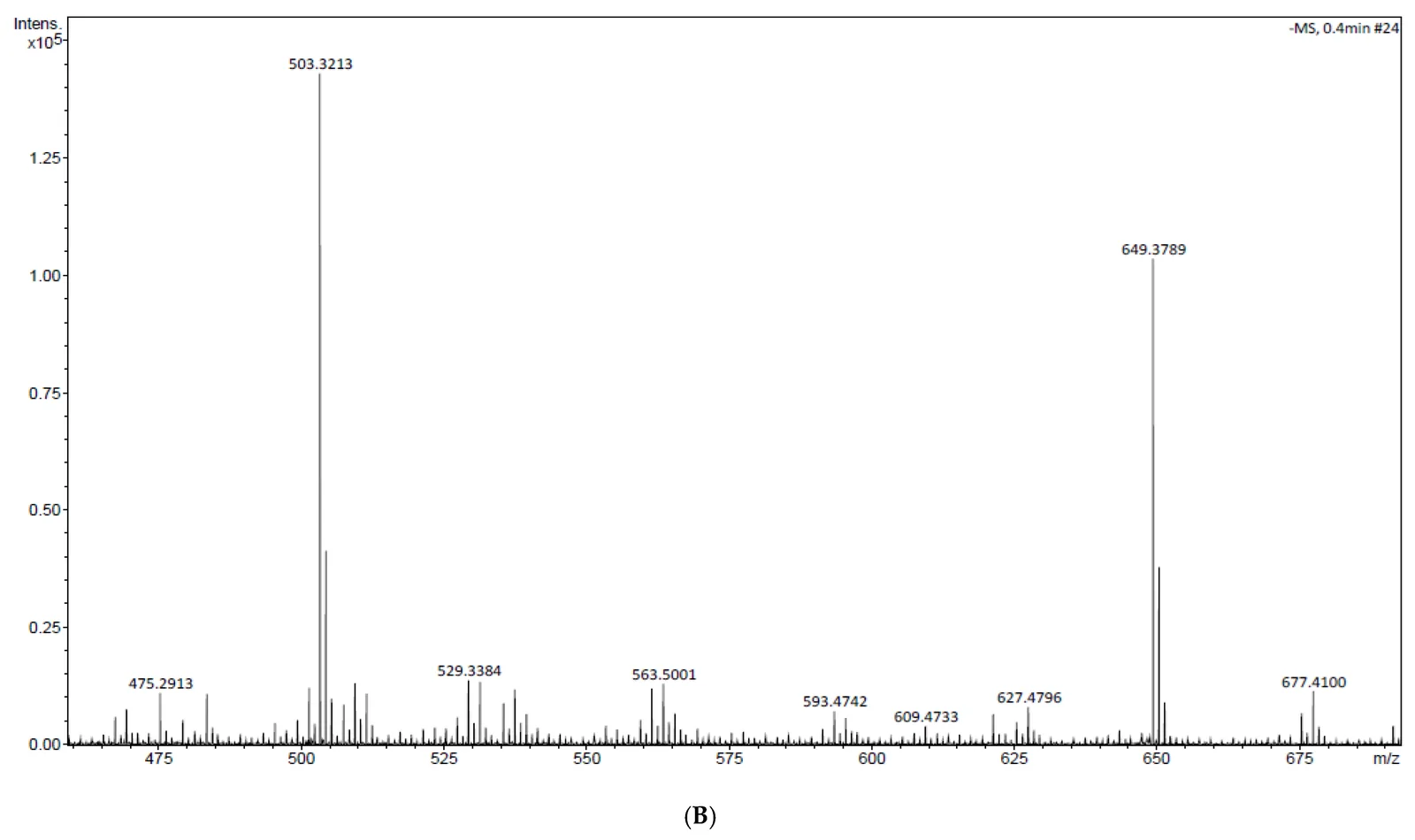
FTIR (**A**) and ESI-MS (**B**) spectra of the RL produced by *P. aeruginosa* LBI in fatty residue.

The RL obtained from the fatty residue was extracted and purity of crude compond estimated by 88%. The product was further analyzed by infrared spectroscopy (FTIR) to identify functional groups characteristic of the biosurfactant. As shown in Figure 3A, the broad band at 3364 cm^−1^ can be attributed to the stretching vibration of hydroxyl (-OH) groups. The symmetric and asymmetric stretching vibrations of the aliphatic C–H bonds, originating from the lipid fraction, are represented by the two intense bands at 2924 and 2855 cm^−1^, respectively. Another characteristic band was observed at 1735 cm^−1^, corresponding to the stretching vibration of carbonyl (C=O) groups present in ester and carboxylic acid moieties. The bands in the 1457–1237 cm^−1^ region can be associated with C–H and O–H deformation vibrations of the carbohydrate moiety. These absorptions are located within the fingerprint region of the spectrum, generally considered to extend from approximately 1460 to 800 cm^−1^. Overall, the FTIR spectrum provides evidence for the glycolipid nature of the extracted biosurfactant and is consistent with previously reported spectra for rhamnolipids [31,42].

Furthermore, the rhamnolipid composition was analyzed by mass spectrometry in negative ion mode (Figure 3B). The RL congeners produced by *P. aeruginosa* LBI using the fatty residue were identified, with RhaC_10_C_10_ (*m/z* 503) and Rha_2_C_10_C_10_ (*m/z* 649) being the predominant RL-related signals, respectively. Nitschke et al. [20] identified ten RL congeners when cultivating the same strain using soybean oil soapstock as the carbon source, also reporting RhaC_10_C_10_ and Rha_2_C_10_C_10_ as predominants. Similar results were observed for *P. aeruginosa* NY3 cultivated in waste frying oil, from which ten RL congeners were identified, with RhaC_10_C_10_ and Rha_2_C_10_C_10_ predominating [43]. In contrast, *Burkholderia thailandensis* E264 cultivated in a food-waste hydrolysate synthesized six RL congeners, with RhaC_14_C_14_, Rha_2_C_12_C_14_, Rha_2_C_14_C_14_, and Rha_2_C_14_C_16_ predominating [44]. As discussed above, factors such as bacterial strain, culture medium composition, cultivation conditions, and culture age influence the final composition of RLs [45].

The critical micelle concentration (CMC) of the RL was determined to be 44.3 mg/L, while the interfacial tension of a 0.1% RL solution against hexadecane was 6.2 mN/m. These results confirm the surfactant properties of the RL produced from the oily residue and support its potential for further applications in industrial and environmental processes.

### 3.3. Detergency Performance Evaluation

**Figure 4:**
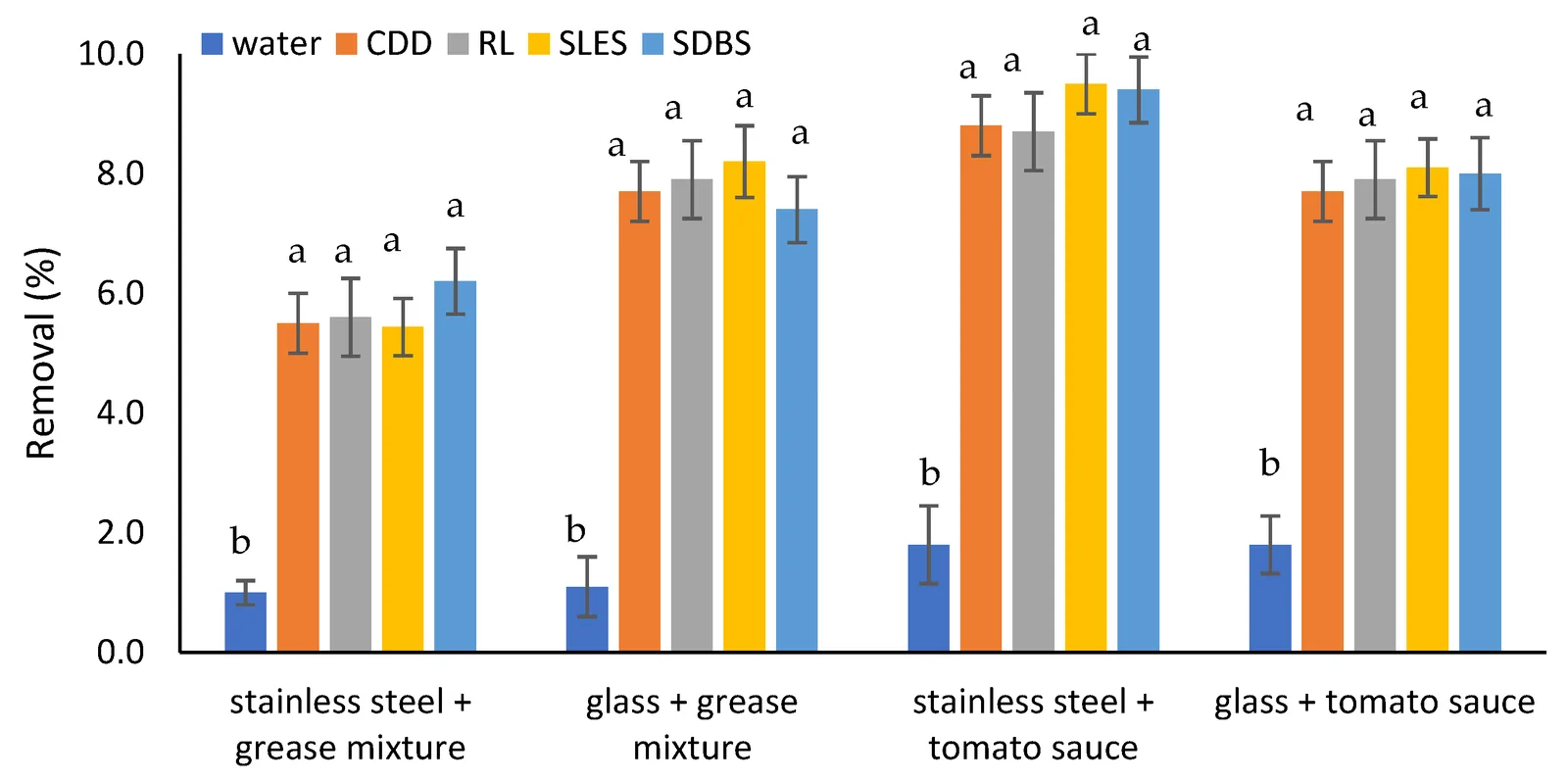
Removal of grease mixture and tomato sauce deposited on the stainless-steel and glass samples during the cleaning assay. CDD: commercial dishwashing detergent; RL: rhamnolipid; SLES: sodium lauryl ether sulfate; and SDBS: sodium dodecylbenzene sulfonate. For each treatment group, bars followed by the same letters do not differ significantly (*p* < 0.05).

The detergency assays compared the performance of the RL produced by *P. aeruginosa* LBI using the fatty residue as a carbon source with those of tap water, a commercial dishwashing detergent (CDD), sodium lauryl ether sulfate (SLES), and sodium dodecylbenzene sulfonate (SDBS), and the results are presented in Figure 4.

For the grease mixture, the RL achieved approximately 8% soil removal under the experimental conditions evaluated. For tomato sauce, the RL also achieved approximately 8% removal from both stainless steel and glass surfaces. The results indicate that statistically significant differences in soil removal were not detected among the different surfactant treatments under the conditions evaluated.

Previous studies have reported higher detergency efficiencies for RLs under different experimental conditions. A detergent formulation containing 40% RL produced by *P. aeruginosa* ATCC 10145 achieved 100% removal of B1 fuel oil from glass and metal surfaces, with performance comparable to those of commercial industrial detergents [28].

Similarly, a 6.4% (*w/v*) RL solution was evaluated against two commercial surfactants, Brij L4 and Triton CG-110, at the same concentration. The RL achieved 63% removal of greasy soil from stainless steel tiles, representing the highest cleaning efficiency among the tested surfactants. According to the authors, this superior performance of RL was associated with the predominance of medium-sized ellipsoidal micelles, characterized by a highly hydrophobic core and low viscosity at the relatively high RL concentration used. These properties may favor the incorporation and removal of hydrophobic soils, making RLs promising candidates for concentrated cleaning formulations [46]. In the present study, the detergency performance of RLs and conventional surfactants suggests that the relatively limited soil removal may have been related, at least in part, to the characteristics of the model soils and the experimental conditions rather than to the intrinsic performance of the surfactants. The composition and adhesion of the model soils, together with the washing conditions employed, may have limited overall soil removal. In addition, the surfactant concentrations used in previous studies reporting higher removal percentages were substantially higher than that used in the present study, which may have contributed to the differences observed. Therefore, these results provide an initial assessment of the detergency potential of the produced rhamnolipids, while further studies using optimized formulations, surfactant concentrations, and application conditions are needed to better evaluate their cleaning performance.

## 4. Conclusions

This study demonstrates the potential of an industrial fatty waste derived from ester synthesis as an alternative substrate for rhamnolipid production by *Pseudomonas aeruginosa* LBI. The efficient utilization of the diverse lipid components of the residue highlights its suitability as a complex carbon source, while the production and characterization of surface-active RLs demonstrate that the proposed substrate can generate a functionally relevant BS. The valorization of this chemical-derived industrial waste for BS production represents a promising sustainable approach that integrates waste valorization with the generation of a value-added bioproduct.

## Figures and Tables

**Table 1:** Composition of fatty residue utilized in this work.

Type	Compound	Proportion (%)
Fatty acids	Lauric acid	4.0
	Myristic acid	7.6
	Palmitic acid	5.5
	Stearic acid	2.5
	Oleic acid	7.2
	Linoleic acid	2.2
Methyl esters	Methyl laurate	1.9
	Methyl palmitate	5.7
	Methyl palmitoleate	0.5
	Methyl stearate	2.4
	Methyl oleate	12.2
	Methyl linoleate	22.1
	Methyl linolenate	2.0
Other esters	Isopropyl myristate	11.0
	Isopropyl palmitate	4.1
	Butyl palmitate	2.9
	Isopropyl oleate	0.7
	Butyl margarate	0.5
	Isobutyl oleate	1.3
	Butyl stearate	3.6

## Data Availability

The original contributions presented in this study are included in the article. Further inquiries can be directed to the corresponding author.
